# Glucocorticoid Dosing and Outcomes in ANCA-Associated Vasculitis With Kidney Involvement

**DOI:** 10.1016/j.ekir.2025.07.022

**Published:** 2025-07-22

**Authors:** Maria Salman, Mark Canney, Ana Maria Naidas, Vimal K. Derebail, Silke R. Brix, Nataliya Milman, Mats L. Junek, Ayub Akbari, Michael Walsh, David Massicotte-Azarniouch

**Affiliations:** 1 Faculty of Medicine, University of Ottawa, Ottawa, Ontario, Canada; 2 Inflammation and Chronic Disease Program, The Ottawa Hospital Research Institute, Ottawa, Ontario, Canada; 3 Division of Nephrology, Department of Medicine, University of Ottawa, Ottawa, Ontario, Canada; 4 Division of Nephrology and Hypertension, University of North Carolina Kidney Center, University of North Carolina at Chapel Hill, Chapel Hill, North Carolina, USA; 5 Division of Cell Matrix Biology and Regenerative Medicine, Manchester University Hospitals NHS Foundation Trust, University of Manchester, Manchester, UK; 6 Division of Rheumatology, Department of Medicine, University of Ottawa, Ontario, Canada; 7 Division of Rheumatology, St. Joseph’s Healthcare, McMaster University, Hamilton, Ontario, Canada; 8 Clinical Epidemiology Program, Ottawa Hospital Research Institute, Ottawa, Ontario, Canada; 9 Department of Medicine, McMaster University, Hamilton, Ontario, Canada; 10 Hamilton Health Sciences, Population Health Research Institute, McMaster University, Hamilton, Ontario, Canada; 11 Kidney Research Centre, The Ottawa Hospital Research Institute, Ontario, Canada

**Keywords:** ANCA-associated vasculitis, death, end-stage kidney disease, glucocorticoids, infection

## Abstract

**Introduction:**

There may be reservations about the guideline-recommended use of reduced glucocorticoid (GC) therapy in severe ANCA-associated vasculitis (AAV). We examined differences in outcomes based on oral GC use during induction therapy for AAV with kidney involvement.

**Methods:**

We conducted a single-center, retrospective cohort study (2010–2023) of patients with biopsy-proven kidney involvement from AAV. Patients were divided into eras 2020 onward (reduced-GC) versus pre-2020 (standard-GC) according to practice shift after adopting a reduced-GC regimen. The primary outcome was the composite of end-stage kidney disease (ESKD) or death within 12 months postbiopsy. Secondary outcomes included serious infection, clinical remission, and change in estimated glomerular filtration rate (eGFR).

**Results:**

There were 138 participants (mean age: 65.2 years, 46.4% female, 68.1% anti-myeloperoxidase), comprising 41 in the reduced-GC era and 97in the standard-GC era. The former group was older (70.3 vs 63.1 years) and had worse baseline kidney function (eGFR: 15.6 vs 22.6 ml/min). The reduction of cumulative GC for reduced-GC compared with the standard-GC era was 39% in the first month, 28% in the first 3 months, and 34% in the first 6 months. For reduced-GC compared with standard-GC era, there was no significant difference in ESKD or death (24.4% vs. 21.6%), serious infection (14.6% vs. 17.5%), or remission (63.4% vs. 63.9%); and both groups showed similar improvement in eGFR over 12 months.

**Conclusion:**

Reduced-GC during induction therapy in individuals with organ-threatening kidney involvement was not associated with a change in outcomes or kidney function recovery. This supports data from a large, randomized trial.

## Graphical abstract

**Figure:**
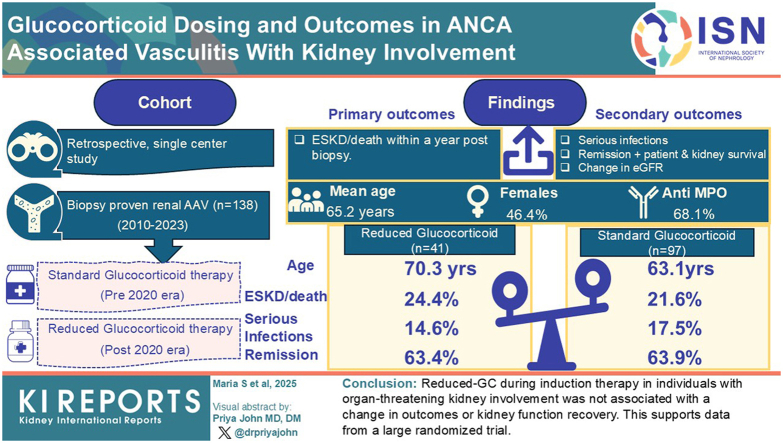

AAV is a group of rare systemic autoimmune disorders that affect the kidneys in approximately 75% of affected individuals and may lead to kidney failure. Even with treatment, about half of patients are left with significant chronic kidney disease and about 20% develop ESKD.^1^^,^^2^ GCs combined with cyclophosphamide or rituximab are the current standard-of-care therapies for AAV with organ- or life-threatening involvement. About 90% of patients achieve remission with these therapies; however, treatment-related adverse events and organ damage from AAV, such as chronic kidney disease, are major contributors to long-term morbidity and mortality.^1^^,^^3^

Clinical trials have demonstrated that reduced-dose GC regimens for induction therapy of AAV can effectively induce remission.^2^^,^^4^ These regimens also lead to fewer infections and decrease GC-related side effects. As such, a reduced-dose GC tapering regimen has become the standard-of-care, guideline-recommended treatment during induction therapy of AAV.^5^^,^^6^ Nevertheless, real-world studies show that clinicians may be reluctant to use reduced-dose GC regimens for AAV, particularly for organ- or life-threatening disease,^7^ and that reduced-GC in severe disease may be associated with worse outcomes compared with standard-GC dosing regimens.^8^ This uncertainty is in spite of results from the Plasma Exchange and Glucocorticoids in Severe ANCA-Associated Vasculitis (PEXIVAS) trial, where reduced-GC was noninferior to a standard-GC regimen in a predominantly severe kidney involvement AAV population.

Our center (The Ottawa Hospital [TOH], Ontario, Canada) adopted the reduced-GC PEXIVAS taper and implemented a change in practice where this regimen became the initial GC-taper used for treatment of AAV with kidney involvement. Since PEXIVAS was published in February 2020,^2^ we examined outcomes in eras of GC use at our center with the year 2020 as the time point to delineate this practice shift. Specifically, our aim was to compare disease and kidney outcomes with GC use in the era of 2020 onward (reduced-GC) to the era of pre-2020 (standard-GC) for the treatment of AAV with biopsy-proven glomerular involvement.

## Methods

### Study Design, Setting and Participants

We performed a retrospective, single-center cohort study of all individuals diagnosed with kidney involvement from AAV confirmed on native kidney biopsy at TOH. The study period was from 1 January 1, 2010 until June 30, 2023 (outcome data collected until July 31, 2024). TOH is a 1200-bed, 3-campus academic teaching hospital, and is the largest tertiary care referral center for adults in a region of > 1.2 million residents. The TOH Glomerulonephritis Clinic Renal Pathology Database was used to identify the study population and baseline characteristics. This database captures all native kidney biopsies performed at TOH, the histopathologic diagnosis, pathology variables, and clinical variables at time of biopsy, and is maintained by a trained clerk. Patients with kidney involvement from AAV are managed in the GC clinic in a shared model, covered by 4 nephrologists who share similar practice habits for the management of AAV.

Participants were individuals who had a first-time kidney biopsy demonstrating pauci-immune glomerulonephritis consistent with AAV (all types of AAV were included). The electronic medical chart was reviewed by the study team to confirm the diagnosis of AAV, and to ascertain exposures and outcomes. There were otherwise no exclusions. Data linkage between the Glomerulonephritis Clinic Renal Pathology Database and the electronic medical chart was completed using medical record numbers. Institutional research and ethics board approval (Ottawa Health Science Network Research Ethics Board) was received before data gathering and study analysis. Owing to the retrospective nature of our study and use of deidentified data, informed consent was waived. The study design, exposure, outcomes, and analysis were all determined before data retrieval. The reporting of this study follows guidelines for observational studies.^9^

### Exposure

The study groups for this before-and-after study were defined by the era of AAV diagnosis. The exposure was the era from 2020 onward (reduced-GC era) and the control was the era pre-2020 (standard-GC era), given the policy shift at our center for reduced-GC use once PEXIVAS results were known.

Patient charts were reviewed to ascertain the type and cumulative dose of induction therapy received for the treatment of AAV (pulse methylprednisolone, prednisone, cyclophosphamide, rituximab, and plasmapheresis). We examined the cumulative dose of prednisone received during induction therapy in the first month, the first 3 months, months 3 to 6, and months 6 to 12 of follow-up. The cumulative dose was determined as best as possible using information from clinic notes and prescribed doses and tapers (we did not have access to outpatient pharmacy prescription data).

### Outcomes

The primary outcome was the composite of ESKD or death within 12 months postbiopsy. ESKD was defined as 12 weeks of chronic dialysis after initial acute dialysis start or initiation of ≥ 12 weeks of chronic dialysis at any time during follow-up. Secondary outcomes included individual components of the composite (ESKD, death) within 12 months, serious infection within 12 months (defined as infection treated with i.v. antibiotics or requiring admission to hospital or leading to death), remission within 6 months (remission defined as when a clinician stated in the clinic note that remission was achieved, or when the clinician transitioned treatment to maintenance therapy for AAV; remission required that neither ESKD nor death had occurred in the first 6 months), change in kidney function over the first 12 months of follow-up, recovery from requirement of acute dialysis (dialysis started within 2 weeks before or after date of biopsy), and relapse within 12 months (based on clinic note documenting new disease manifestations requiring intensification of therapy after having previously achieved remission).

### Analysis

The date of biopsy was the index date for start of follow-up. Participants were followed up with until death, kidney transplant, or last nephrology clinic visit. Baseline characteristics, exposures, and outcomes are presented for the total cohort and by era of GC use (pre-2020: standard-GC era; 2020 onward: reduced-GC era). Descriptive statistics, means with SDs, medians with interquartile ranges, and counts with percentages were presented along with appropriate statistic. Time-to-event analysis and Cox proportional hazards models were calculated for the composite outcome (ESKD or death) by era of GC use, using standard-GC as the reference. Crude and adjusted hazard ratios were calculated with 95% confidence intervals; models were adjusted for age and kidney function at biopsy after noting a marked difference in these baseline characteristics between the exposure groups. We used generalized linear mixed models, adjusting for age and eGFR at time of biopsy, to compare change in eGFR over 12 months postbiopsy in the groups. Least square means differences in change in eGFR between the 2 groups, with 95% confidence interval, were calculated at 1, 3, 6, and 12 months postbiopsy. Serum creatinine values were censored after the development of ESKD, and no imputation was done for missing data, such that an individual’s creatinine values were used until ESKD or end of follow-up. Serum creatinine values at biopsy and at 1, 3, 6, and 12 months postbiopsy were converted to eGFR using the Chronic Kidney Disease-Epidemiology Collaboration 2021 formula.^10^

We performed the following additional analyses: (i) association of the tertiles of cumulative prednisone use in the first month postbiopsy, because this is when patients typically receive the highest doses of prednisone, with serious infection and with the primary outcome within 12 months (tertiles were chosen in order to have sufficient group sizes for comparison); (ii) outcomes by era of GC use, stratified by type of induction agent used (rituximab or cyclophosphamide); statistical tests were not carried out for the analysis stratified by induction agent because it would serve more of a descriptive purpose due to the low number of patients and events expected in subcategories; and (iii) results for the primary outcome and serious infection after reclassifying individuals in the standard-GC era who actually received reduced-GC during induction (our center recruited for the PEXIVAS trial during the study period and some patients received reduced-GC taper as per PEXIVAS) as being reduced-GC.

All analyses were performed with SAS software (v9.4, SAS Institute). *P*-values < 0.05 were considered statistically significant.

## Results

### Baseline Characteristics and Induction Therapies

**Figure 1:**
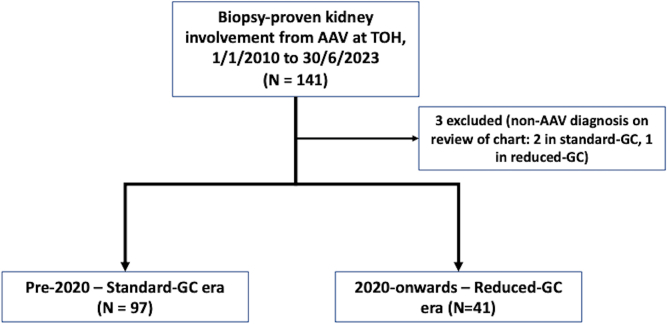
Patient flow chart. AAV, antineutrophil cytoplasmic antibody–associated vasculitis, GC, glucocorticoid; TOH, The Ottawa Hospital.

There were 141 patients identified with a biopsy-proven first diagnosis of AAV, with 3 patients excluded because review of the chart revealed diagnoses other than pauci-immune ANCA-associated GN (2 in standard-GC era, 1 in reduced-GC). The study population consisted of 138 patients, 97 in the standard-GC era and 41 in the reduced-GC era (Figure 1). The median (interquartile range) follow-up for the study cohort was 4.1 (1.7–7.5) years. Participants in the reduced-GC era were older (mean age: 70.3 vs. 63.1 years) and had worse kidney function at time of biopsy (median serum creatinine: 334 vs 237 μmol/l) compared with standard-GC era, respectively. Baseline characteristics are shown in Table 1. The induction therapies used are summarized in Table 2. The vast majority of patients, in both eras, received methylprednisolone pulses (median: 1500 mg total). There was less prednisone used in reduced-GC era than in the standard-GC over the first 6 months postbiopsy: cumulative dose in the first 6 months were 2747 mg versus 4175 mg, respectively (34% reduction overall; 39% reduction in month 1, 28% reduction in months 0–3, and 51% reduction in months 3–6). Eleven patients had removal of prednisone in the first 6 months (5 in reduced-GC, 6 in standard-GC). For the reduced-GC era compared with the standard-GC era, rituximab-only induction was more common (43.9% vs. 10.3%, respectively) and cyclophosphamide-only induction was less common (39.0% vs. 78.4%, respectively). No individuals in either the standard-GC or reduced-GC era received avacopan. Trimethoprim-sulfamethoxazole was routinely used for *Pneumocystis jiroveci* prophylaxis in both GC eras.

**Table 1:** Baseline characteristics

Characteristics	Total (138)	Standard-GC era (97)	Reduced-GC era (41)
Age; yrs, mean (SD)	65.2 (14.2)	63.1 (15.2)	70.3 (10.2)
Sex; *n* (%)			
Female	64 (46.4)	45 (46.4)	19 (46.3)
Male	74 (53.6)	52 (53.6)	22 (53.7)
Serum creatinine at biopsy, μmol/l; median (IQR)	245 (168–414)	237 (163–390)	334 (185–483)
Acute dialysis requirement; *n* (%)	26 (18.8)	16 (16.5)	10 (24.4)
ANCA Berden class; *n* (%)			
Focal	60 (47.6)	39 (45.4)	21 (52.5)
Diffuse	19 (15.1)	12 (14.0)	7 (17.5)
Mixed	35 (27.8)	25 (29.1)	10 (25.0)
Sclerotic	12 (9.5)	10 (11.6)	2 (5.0)
% normal glomeruli; median (IQR)	48 (34–68)	47 (34–63)	50 (34–75)
IFTA grade; *n* (%)			
None/minimal	61 (46.6)	42 (44.7)	19 (51.4)
Mild	44 (33.6)	34 (36.2)	10 (27.0)
Moderate	13 (9.9)	9 (9.6)	4 (10.8)
Severe	13 (9.9)	9 (9.6)	4 (10.8)
ANCA type; *n* (%)			
MPO	94 (68.1)	66 (68.0)	28 (68.3)
PR3	46 (33.3)	32 (33.0)	14 (34.2)

GC, glucocorticoid; IFTA, interstitial fibrosis and tubular atrophy; IQR, interquartile range; MPO, myeloperoxidase; PR3, proteinase 3.

Some variables do not add up to column totals owing to missing values.

**Table 2:** Induction therapies

Therapies	Total (138)	Standard-GC era (97)	Reduced-GC era (41)
Methylprednisolone pulse			
*n* (%)	110 (79.7)	80 (82.5)	30 (73)
Dose, mg; median (IQR)	1500 (1100–1500)	1500 (1000–1500)	1500 (1500–1500)
Prednisone cumulative, mg^a^; median (IQR)			
0–1 mo	1400 (980–1800)	1605 (1225–1820)	980 (805–1225)
0–3 mo	2730 (1960–4310)	3120 (2310–4500)	2235 (1830–2491)
3–6 mo	840 (475–1440)	1055 (671–1920)	512 (450–603)
6–12 mo	905 (660–1103)	910 (825–1350)	900 (455–910)
Cyclophosphamide only; *n* (%)	92 (66.7)	76 (78.4)	16 (39.0)
Rituximab only; *n* (%)	28 (20.3)	10 (10.3)	18 (43.9)
Cyclophosphamide and rituximab; *n* (%)	8 (5.8)	3 (3.1)	5 (12.2)
Plasmapheresis; *n* (%)	22 (16.1)	22 (22.7)	0

GC, glucocorticoid; IQR, interquartile range.

Totals do not always add up to 100% because of missing data/loss to follow-up.

a Does not include methylprednisolone.

### Full Cohort Outcomes

Throughout the full study follow-up, the composite of ESKD or death occurred in 37.7% of patients; ESKD in 18.8%, death in 25.4%, and a serious infection in 26.7%. Remission at 6 months was achieved in 63.8% of patients, 26.1% required acute dialysis (25% of whom recovered), and 19.5% sustained a relapse. The eGFR improved over the first year, with most improvement happening in the first 3 months (Supplementary Figure S1).

### ESKD and Death by GC Era

**Figure 2:**
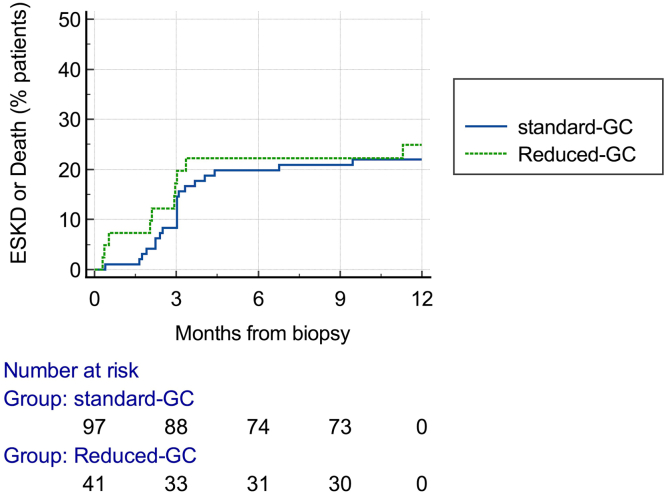
Survival analysis of time to composite outcome (ESKD or death). ESKD, end-stage kidney disease; GC, glucocorticoid.

In the first 12 months, for the eras of reduced-GC versus standard-GC, there was no significant difference in the proportion of patients with ESKD or death (24.4% vs. 21.6%, *P* = 0.72), ESKD (12.2% vs. 15.5%, *P* = 0.61), or death of any cause (14.6% vs. 9.3%, *P* = 0.35). Time-to-event analysis for the first 12 months did not demonstrate any significant difference in the rate (Figure 2) or risk of developing ESKD or death (adjusted hazard ratio: 0.92 [0.42–2.00]; standard-GC era referent) (Table 3).

**Table 3:** Outcomes by era of GC use (in first 12 months)

Outcomes	Standard-GC era (97)	Reduced-GC era (41)	*P*-value
ESKD or death; *n* (%)	21 (21.6)	10 (24.4)	0.72
Crude HR (95% CI)	Reference	1.20 (0.57–2.55)	
Adjusted HR (95% CI)	Reference	0.92 (0.42–2.00)	0.83
ESKD; *n* (%)	15 (15.5)	5 (12.2)	0.61
Death; *n* (%)	9 (9.3)	6 (14.6)	0.36
Serious infection; *n* (%)	17 (17.5)	6 (14.6)	0.68
Remission at 6 mo; *n* (%)	62 (63.9)	26 (63.4)	0.96
Recovery from dialysis dependence; *n* (%)	4/16 (25.0)	5/10 (50.0)	0.19

CI, confidence interval; ESKD, end-stage kidney disease; GC, glucocorticoid; HR, hazard ratio.

### Secondary Outcomes by GC Era

**Figure 3:**
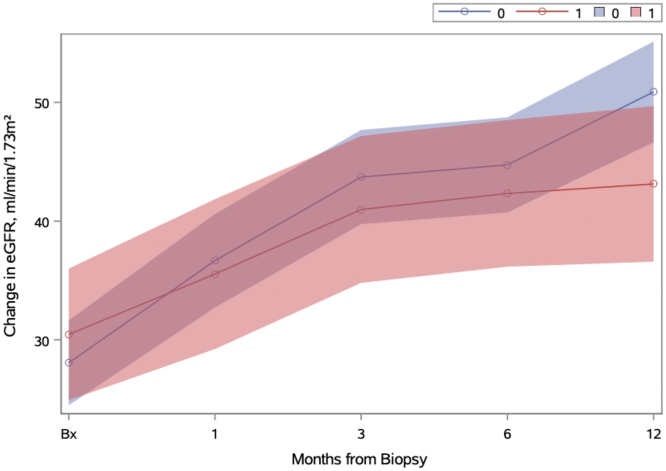
Change in eGFR (mean and 95% CI) over the first 12 months, by era of GC use. 0, standard-GC; 1, reduced-GC; adjusted for age and serum creatinine at biopsy. CI, confidence interval; eGFR, estimated glomerular filtration rate; GC, glucocorticoid.

For the eras of reduced-GC versus standard-GC, serious infection within 12 months occurred in 14.6% versus 17.5% (*P* = 0.68). Both groups had similar rates of remission within 6 months (63.9% vs. 63.4%, *P* = 0.96) (Table 3); the reasons for not having remission were ESKD or death within 6 months (22% vs. 21%) and remission occurring at 6 to 12 months (15% vs. 10%). Recovery from acute dialysis occurred in 5 of 10 in the reduced-GC era compared with 4 of 16 patients in the standard-GC era (*P* = 0.19). Both groups showed similar improvement in eGFR after biopsy with a nonstatistically significant greater improvement in standard-GC era versus reduced-GC at 12-months (ΔGFR difference and 95% confidence interval: 7.7 [−0.1 to 15.6]) (Figure 3 and Table 4).

**Table 4:** eGFR ml/min per 1.73 m^2^ (means and 95% CI) over 12 months from biopsy, adjusted for baseline age and eGFR

Time point	Standard-GC era	Reduced-GC era	ΔGFR standard minus ΔGFR reduced	*P*-value (standard minus reduced)
Biopsy	28.1 (24.5–31.6)	30.4 (24.9–36.0)	-	-
1 mo	36.7 (32.7–40.6)	35.5 (29.2–41.8)	1.1 (−6.3 to 8.6)	0.76
3 mo	43.7 (39.7–47.7)	41.0 (34.8–47.2)	2.7 (−4.6 to 10.1)	0.47
6 mo	44.7 (40.7–48.7)	42.3 (36.2–48.5)	2.4 (−5.0 to 9.8)	0.52
12 mo	50.9 (46.6–55.1)	43.1 (36.6–49.7)	7.7 (−0.1 to 15.6)	0.05

ΔGFR, change in GFR, CI, confidence interval; eGFR, estimated glomerular filtration rate, GC, glucocorticoid.

### Additional Analyses

In Table 5, we show results of the analysis by tertiles of cumulative prednisone in the first month. Eight patients were excluded from this analysis because they did not receive any methylprednisolone pulses or prednisone in the first month after biopsy. There were 43 patients in the lowest (median: 875mg), 41 in the middle (median: 1435mg) and 46 in the highest tertile (median: 1838 mg). Serious infections occurred in 9.3%, 22.0%, and 21.7%, respectively. The composite ESKD or death occurred in 25.6% versus 29.3% versus 15.2%, respectively; and there was no significant difference in the risk of ESKD or death in the lowest tertile compared with the highest tertile (crude hazard ratio: 1.77 [95% confidence interval: 0.66–4.75]).

**Table 5:** Outcomes in first year by tertiles of total prednisone use in the first month

Outcomes	Tertile 1 (43)	Tertile 2 (41)	Tertile 3 (46)
Cumulative prednisone 1^st^ mo; median (IQR)	875 (645–980)	1435 (1225–1540)	1838 (1800–2125)
Induction therapy, *n* (%)			
Methylprednisolone pulse	33 (76.7)	37 (90.2)	38 (82.6)
Rituximab	20 (46.5)	8 (19.5)	7 (15.2)
Cyclophosphamide	21 (48.8)	37 (90.2)	39 (84.8)
eGFR at baseline, ml/min; median (IQR)	21.6 (10.3–36.6)	18.5 (10.6–27.0)	25.4 (13.2–41.9)
ESKD or death first 12 mo; *n* (%)	11 (25.6)	12 (29.3)	7 (15.2)
Crude HR (95% CI)	1.77 (0.66–4.75)	2.49 (0.94–6.63)	Reference
Serious infection first 12 mo; *n* (%)	4 (9.3)	9 (22.0)	10 (21.7)

CI, confidence interval; eGFR, estimated glomerular filtration rate; ESKD, end-stage kidney disease; HR, hazard ratio; IQR, interquartile range.

When examining participants treated with rituximab only compared with those treated with cyclophosphamide only, ESKD or death occurred in 4 of 28 (14.8%) versus 22 of 92 (23.9%), respectively. Stratified by era of GC use, for rituximab only versus cyclophosphamide only, ESKD or death respectively occurred in 20.0% versus 19.7% in the standard-GC era and in 11.8% versus 43.8% in the reduced-GC era (Supplementary Table S1).

There were 7 participants in the standard-GC era who received initial reduced-GC prednisone taper because they participated in the PEXIVAS trial. Two such participants had ESKD or death within 12 months, and none had serious infection. When reclassifying these 7 individuals as reduced-GC, there was no major change in rates of ESKD or death, or of serious infection compared with the primary analysis (Supplementary Table S2). All patients in the reduced-GC era were initially given reduced-GC prednisone taper as per PEXIVAS trial.

## Discussion

In our study, we investigated different eras of practice for GC use in a real-world scenario for patients with biopsy-proven kidney involvement from AAV. When comparing standard-GC versus reduced-GC use as determined by eras pre- and post-PEXIVAS, we found that reduced-GC era was not associated with worse outcomes. There was no significant difference in the risk for the composite ESKD or death, or for each individual component. There were similar rates of serious infection and remission, and both groups showed similar improvement in eGFR. These findings corroborate those from the PEXIVAS trial that reduced GCs are a safe and effective option for induction treatment in a patient population predominantly presenting with severe kidney involvement.

At the time of writing, PEXIVAS is the largest study conducted in AAV and has had practice-changing impact. At our center, we fully adopted the findings from PEXIVAS, which created a natural time point for the before-and-after experiment, forming the basis for the main study exposure. Our patients treated from 2020 onward had similar levels of total GC exposure and reduction compared with pre-2020 as what was achieved in the PEXIVAS reduced-GC arm. The patient populations in ours and the PEXIVAS study were similar in terms of baseline characteristics, initial disease severity, and in the strikingly high prevalence of ESKD and death incurred. This highlights the importance of intervening on factors that could improve recovery of kidney function and reduce mortality in patients with severe kidney involvement from AAV.

In both the reduced-GC and standard-GC eras, the improvement in renal function was greatest in the first 3 months, and both continued to improve up to the 12-month mark. Kidney function improvement was greater in standard-GC from 6 months onward, but not significantly so, consistent with what was seen in PEXIVAS.^11^ It is difficult to determine whether the greater improvement in kidney function after 6 months in standard- versus reduced-GC in our study is simply a chance finding because the cumulative dose of prednisone was essentially the same between the 2 groups from 6 to 12 months. One explanation may be that early effects from induction therapy could have an impact on long-term kidney function, as has been demonstrated with plasmapheresis.^2^^,^^11^^,^^12^ Finding ways to enhance recovery of kidney function is of the utmost importance to improve outcomes in AAV, given the association of chronic kidney disease with infection and cardiovascular disease, which are major contributors to short- and long-term mortality in AAV.^3^^,^^13^^,^^14^ Considering this, the results from the Avacopan for the Treatment of ANCA-Associated Vasculitis trial are of interest, where avacopan showed greater improvements in eGFR versus prednisone, particularly in patients with low eGFR.^15^^,^^16^ Although these results are promising, further investigation through a dedicated trial specifically examining recovery of kidney function in patients with severe kidney impairment is needed to form more solid conclusions.

Patients with severe AAV carry a high burden of infections and these are a leading cause of death in the first year after diagnosis of AAV.^2^^,^^3^ Both the PEXIVAS and the Effect of Reduced-Dose versus High-Dose Glucocorticoids Added to Rituximab on Remission Induction in ANCA-Associated Vasculitis trials demonstrated that reduced GC use was associated with fewer infections, in what seems to be a dose-dependent manner.^2^^,^^4^ Though we did not demonstrate a significant difference in serious infections in our main analysis, patients who received the lowest amount of prednisone in the first month had the lowest rates of serious infections. However, this group showed a trend to more ESKD or death compared with the highest prednisone use in the first month. This highlights the importance of finding GC-sparing induction regimens that could minimize GC use in patients with severe kidney involvement, without reducing treatment efficacy. Observational data suggest that as little as 1 to 2 weeks of GC, when combined with cyclophosphamide and rituximab, may be used in patients with severe AAV without compromising disease control.^17^ Such a regimen would benefit from being studied in a prospective fashion.

Nagle *et al.* published real-world experience from the French Vasculitis Study Group with reduced-GC compared with standard-GC in patients with severe AAV.^8^ They found that reduced-GC had worse outcomes (composite ESKD, death, disease progression, and relapse) than standard-GC. A possible explanation for these contrasting results may be differences in patient population. A rheumatology-predominant population may have more extrarenal manifestations, which could worsen with rapid tapering of prednisone. We did not capture the number or extent of extrarenal manifestations in our study; however, from experience, most of our patients present with predominantly kidney-limited disease. In the study by Nagle *et al.*, 70% had kidney involvement with a median baseline creatinine of 134 μmol/l and 8.5% requiring acute dialysis, which is less severe than in our study (baseline creatinine of 245 μmol/l and 18.8% requiring acute dialysis). Nevertheless, they found an increased risk for a composite of ESKD or death with reduced-GC whereas we did not. This difference is more difficult to reconcile but could relate to the type of induction agent used. We did not find a signal for worse outcomes among rituximab-treated patients with reduced-GC compared with standard-GC or compared with cyclophosphamide-treated patients with reduced-GC, but only 44% of patients in our study received rituximab-only induction; therefore, the small sample size and number of events in these subgroups limit the interpretation of these findings. AAV is a heterogenous disease, and patient-specific management based on disease manifestation may be appropriate. Some patients may not warrant a rapid reduction in GC. Nonetheless, in a predominantly severe kidney involvement population, our findings corroborate those from PEXIVAS and suggest that reduced GC is a safe effective option.

Findings from our study should be taken in the context of a unique global event that occurred shortly after publication of the PEXIVAS trial. The COVID-19 pandemic was at its peak from 2020 to 2022, which overlapped with most of our reduced-GC era. The impact of the pandemic, with global lockdowns and redirecting of hospital and medical resources, on patient care is difficult to quantify. However, it is reasonable to consider that it could have led to delays in medical evaluation, specialist referral and availability, and diagnosis of AAV. This could account for worse baseline kidney function and increased acute dialysis requirement in the reduced-GC group in our study. Patient care may have suffered through less frequent or less optimal (virtual) medical follow-ups, which could have negatively affected patient outcomes. Patients may have suffered more serious infections from COVID-19; however, lockdowns could have limited the ability to ascertain certain non-COVID-19 serious infections. Ultimately, the potential impacts of the pandemic are unclear. Future studies may help elucidate whether this factor has had an impact on the safety and efficacy of reduced-GC regimen, because present day patient care is less affected by COVID-19 protocols.

The strengths of our study include generalizability and granularity of data. Though the results were based on a single center, the care and practice is likely reflective of other GN centers in Ontario and Canada because of similar drug availability and overall adoption of the PEXIVAS reduced-GC regimen. All our patients had biopsy-proven glomerular involvement from ANCA-associated vasculitis, and by performing in-depth chart review, we obtained comprehensive information on GC exposure. Our study has several limitations. First, as a single-centered study, sample size limits our ability to draw firm conclusions, particularly for certain subgroup analyses. Second, grouping patients by era of GC use means that we are not necessarily comparing exact patient-level dosages of GC. We chose eras because patients may receive different amounts of GC than initially intended due to clinical evolution (disease control and complications). Our analysis by tertiles of prednisone use in the first month compares more exact measures of GC exposure and found similar findings to our main analysis. Third, some outcomes such as serious infections may have been missed because of patients presenting to peripheral hospitals. This should affect both groups equally, biasing toward the null, but may explain the lower rates of serious infections in our study than in the PEXIVAS trial. Fourth, we did not capture data on severe nonkidney manifestations of AAV, such as uveitis, alveolar hemorrhage, or mono-neuritis multiplex, which prevented us from analyzing the impact of reduced-GC use at our center on these manifestations. Fifth, our definition of remission was not based on specific markers of disease activity or traditional definitions for vasculitis trials using the Birmingham Vasculitis Activity Score for Granulomatosis with Polyangiitis. We do not routinely use this tool, and it has has important limitations which make it difficult to implement in routine practice. Our definition may be more reflective of what is done in clinical practice outside of dedicated vasculitis centers. Finally, owing to the observational nature of this before-and-after study, there is the risk of residual confounding such as other changes in patient care that could have occurred over time (e.g., COVID-19 impact), which may have affected outcomes.

In conclusion, this cohort study in a non-trial setting showed that a practice of reduced-GC for biopsy-proven kidney involvement from AAV was not associated with worse outcomes. Our findings reinforce the safety and efficacy of reduced-GC as the initial GC regimen of choice for patients with severe kidney involvement. To improve patient outcomes and reduce treatment-related complications, future studies should explore strategies to further reduce-GC exposure, reduce rates of infection, and improve kidney function recovery.

## Disclosure

NM has received consultancy fees from Boehringer Ingelheim, AbbVie, Fresenius Kabi, Otsuka, and AstraZeneca; and speaker fees from Boehringer Ingelheim, Fresenius Kabi, and GSK. VKD reports consultancy for Novartis, Travere Therapeutics, Bayer, Forma Therapeutics, Merck, Amgen, iCell Gene Therapeutics, and Vera Therapeutics; and honoraria from UpToDate. All the other authors declared no competing interests.

### Funding

This study was supported by a 10.13039/100007211Vasculitis Foundation Young Investigator Program Grant to DMA (# VF_Massicotte-Azarniouch.4.2023) and by an unrestricted grant from Otsuka to AA. The funders had no role in the design, conduct or interpretation of results of this study.

## Data Availability Statement

Data cannot be shared publicly because of ethical concerns and privacy restrictions in accordance with our institution's ethics board. Data requests can be directed to the Ottawa Health Sciences Network Research Ethics Board (rebadministration@ohri.ca).

## Author Contributions

Study conception and design were by MC, AMN, VKD, SRB, MW, and DM-A. Data retrieval was by MS, AMN, and DM-A. Statistical analysis was by AA. Writing of first draft was by MS and DM-A. All coauthors interpreted the results, provided critical revision and editing of the manuscript, and provided important intellectual content.

## Supplementary Material

Supplementary File (PDF) **Figure S1.** Change in median estimated glomerular filtration rate in first year, full study cohort. **Table S1.** Outcomes in the first 12 months, by type of induction therapy. **Table S2.** Outcomes by glucocorticoid use (in first 12 months), after reclassification of 7 patients. STROBE Checklist.

## Footnotes

Supplementary File (PDF)
**Figure S1.** Change in median estimated glomerular filtration rate in first year, full study cohort. **Table S1.** Outcomes in the first 12 months, by type of induction therapy. **Table S2.** Outcomes by glucocorticoid use (in first 12 months), after reclassification of 7 patients. STROBE Checklist.
