# Supplementary material for: Glucocorticoid Dosing and Outcomes in ANCA-Associated Vasculitis With Kidney Involvement
Source: Kidney Int Rep. 2025 Jul 22;10(10):3456–64. doi: 10.1016/j.ekir.2025.07.022 (PMC12545648; doi:10.1016/j.ekir.2025.07.022)
Supplement: Supplementary File (PDF) — Figure S1. Change in median estimated glomerular filtration rate in first year, full study cohort. Table S1. Outcomes in the first 12 months, by type of induction therapy. Table S2. Outcomes by glucocorticoid use (in first 12 months), after reclassification of 7 patients. STROBE Checklist. [file mmc1.pdf]

Supplemental Material

STROBE Statement—checklist of items that should be included in reports of observational studies

	Item No.	Recommendation	Page No.	Relevant text from manuscript
**Title and abstract**	1	(*a*) Indicate the study's design with a commonly used term in the title or the abstract	1	Title
		(*b*) Provide in the abstract an informative and balanced summary of what was done and what was found	3	see abstract
**Introduction**				
Background/rationale	2	Explain the scientific background and rationale for the investigation being reported	4	See Introduction section
Objectives	3	State specific objectives, including any prespecified hypotheses	4	"our aim was to compare disease and kidney outcomes with GC use in the era of 2020-onwards (reduced-GC) to the era of pre-2020 (standard-GC) for the treatment of AAV with biopsy-proven glomerular involvement"
**Methods**				
Study design	4	Present key elements of study design early in the paper	5	"We performed a retrospective, single-centre cohort study…"
Setting	5	Describe the setting, locations, and relevant dates, including periods of recruitment, exposure, follow-up, and data collection	5	"…all individuals diagnosed with kidney involvement from AAV confirmed on native kidney biopsy at The Ottawa Hospital (TOH). The study period was from 1 January 2010 until 30 June 2023 (outcome data collected until 31 July 2024)"
Participants	6	(*a*) *Cohort study*—Give the eligibility criteria, and the sources and methods of selection of participants. Describe methods of follow-up<br>*Case-control study*—Give the eligibility criteria, and the sources and methods of case ascertainment and control selection. Give the rationale for the choice of cases and controls<br>*Cross-sectional study*—Give the eligibility criteria, and the sources and methods of selection of participants	5	"Participants were individuals who had a first time kidney biopsy demonstrating pauci-immune glomerulonephritis consistent with AAV (all types of AAV were included). The electronic medical chart was reviewed by the study team to confirm the diagnosis of AAV, ascertain exposures and outcomes. There were otherwise no exclusions"

		(*b*) *Cohort study*—For matched studies, give matching criteria and number of exposed and unexposed<br><br>*Case-control study*—For matched studies, give matching criteria and the number of controls per case	N/A	
Variables	7	Clearly define all outcomes, exposures, predictors, potential confounders, and effect modifiers. Give diagnostic criteria, if applicable	5, 6	Exposure paragraph<br><br>Outcomes paragraph
Data sources/ measurement	8*	For each variable of interest, give sources of data and details of methods of assessment (measurement). Describe comparability of assessment methods if there is more than one group	5,6	"The electronic medical chart was reviewed by the study team to confirm the diagnosis of AAV, ascertain exposures and outcomes"
Bias	9	Describe any efforts to address potential sources of bias	7	Additional analyses<br><br>"…results for the primary outcome and serious infection after re-classifying individuals in the standard-GC era who actually received reduced-GC during induction (our centre recruited for the PEXIVAS trial during the study period and some patients received reduced-GC taper as per PEXIVAS) as being reduced-GC"
Study size	10	Explain how the study size was arrived at	5	"…single-centre cohort study of all individuals diagnosed with kidney involvement from AAV confirmed on native kidney biopsy at The Ottawa Hospital (TOH)"

Continued on next page

Quantitative variables	11	Explain how quantitative variables were handled in the analyses. If applicable, describe which groupings were chosen and why	6	"Patient charts were reviewed to ascertain the type and cumulative dose of induction therapy received for the treatment of AAV (pulse methylprednisolone, prednisone, cyclophosphamide, rituximab, plasmapheresis). We examined the cumulative dose of prednisone received during induction therapy in the first month, the first 3 months, months 3-6 and months 6-12 of follow-up. The cumulative dose was determined as best as possible using information from clinic notes and prescribed doses and tapers"
Statistical methods	12	(*a*) Describe all statistical methods, including those used to control for confounding	6, 7, 8	"…models were adjusted for age and kidney function at biopsy…"
		(*b*) Describe any methods used to examine subgroups and interactions	7	"outcomes by era of GC use, stratified by type of induction agent used (rituximab, cyclophosphamide). Statistical tests were not carried out for the analysis stratified by induction agent since it would serve more of a descriptive purpose due to the low number of patients and events expected in sub-categories"
		(*c*) Explain how missing data were addressed	7	"Serum creatinine values were censored after the development of ESKD, and no imputation was done for missing data"
		(*d*) *Cohort study*—If applicable, explain how loss to follow-up was addressed<br>*Case-control study*—If applicable, explain how matching of cases and controls was addressed<br>*Cross-sectional study*—If applicable, describe analytical methods taking account of sampling strategy	6	"Participants were followed until death, kidney transplant or last nephrology clinic visit"
		(*e*) Describe any sensitivity analyses	7	Additional analyses
**Results**				
Participants	13*	(a) Report numbers of individuals at each stage of study—eg numbers potentially eligible, examined for eligibility, confirmed eligible, included in the study, completing follow-up, and analysed	8	Results<br>Baseline characteristics and induction therapies paragraph

		(b) Give reasons for non-participation at each stage	8	"…three patients excluded because review of the chart revealed diagnoses other than pauci-immune ANCA-associated GN (2 in standard-GC era, 1 in reduced-GC)"
		(c) Consider use of a flow diagram	Figure 1	Figure 1
Descriptive data	14*	(a) Give characteristics of study participants (eg demographic, clinical, social) and information on exposures and potential confounders	8	Results<br>Baseline characteristics and induction therapies paragraph<br>Table 1<br>Table 2
		(b) Indicate number of participants with missing data for each variable of interest	N/A	N/A
		(c) *Cohort study*—Summarise follow-up time (eg, average and total amount)	Not reported	Not reported
Outcome data	15*	*Cohort study*—Report numbers of outcome events or summary measures over time	8, 9	Results<br>Full study cohort section
		Case-control study—Report numbers in each exposure category, or summary measures of exposure	N/A	
		Cross-sectional study—Report numbers of outcome events or summary measures	N/A	
Main results	16	(*a*) Give unadjusted estimates and, if applicable, confounder-adjusted estimates and their precision (eg, 95% confidence interval). Make clear which confounders were adjusted for and why they were included	9, 10	Results<br>ESKD and Death by GC era section<br>Secondary outcomes by GC era section<br>Table 3
		(*b*) Report category boundaries when continuous variables were categorized	N/A	
		(*c*) If relevant, consider translating estimates of relative risk into absolute risk for a meaningful time period	N/A	

Continued on next page

Other analyses	17	Report other analyses done—eg analyses of subgroups and interactions, and sensitivity analyses	9, 10	Results<br><br>Additional analyses section
**Discussion**				
Key results	18	Summarise key results with reference to study objectives	10	Discussion<br><br>First paragraph
Limitations	19	Discuss limitations of the study, taking into account sources of potential bias or imprecision. Discuss both direction and magnitude of any potential bias	13, 14	Discussion<br><br>"Our study has several limitations…"
Interpretation	20	Give a cautious overall interpretation of results considering objectives, limitations, multiplicity of analyses, results from similar studies, and other relevant evidence	10, 11, 12, 13	Discussion
Generalisability	21	Discuss the generalisability (external validity) of the study results	14	Discussion
**Other information**				
Funding	22	Give the source of funding and the role of the funders for the present study and, if applicable, for the original study on which the present article is based	1	"This study was supported by a Vasculitis Foundation Young Investigator Program Grant to DMA (# VF_Massicotte-Azarniouch.4.2023) and by an unrestricted grant from Otsuka to AA. The funders had no role in the design, conduct or interpretation of results of this study."

Figure S1. eGFR (median) in first year, full study cohort

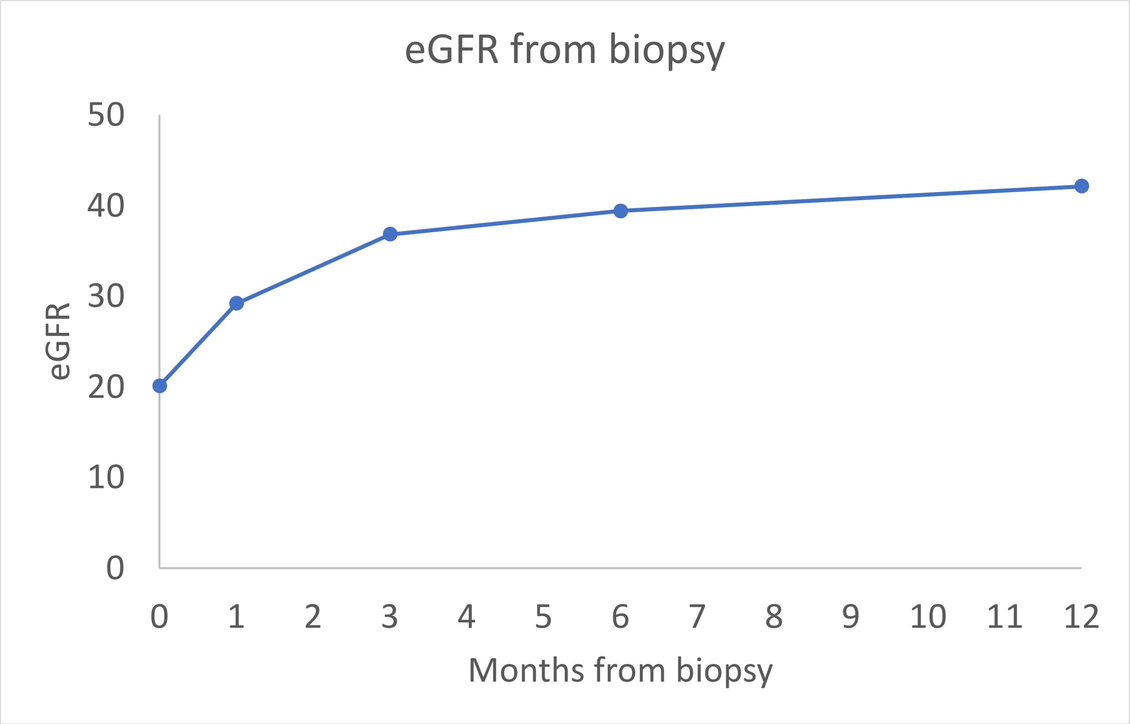

eGFR median (IQR)

Biopsy	1-month	3-month	6-month	12-month
20 (11, 38)	29 (19, 44)	37 (26, 58)	39 (27, 58)	42 (31, 66)

Table S1. Outcomes in first 12 months, by type of induction therapy

	**Rituximab only (28)**	**Cyclophosphamide only (92)**	**Rituximab + Cyclophosphamide (8)**
Age; mean (SD)	61 (16.9)	66.5 (12.5)	69.5 (17.5)
eGFR at biopsy; median (IQR)	21.3 (12.5, 41.9)	20.7 (10.6, 35.3)	9.8 (8.2, 28.0)
ESKD or death; N (%)	4 (14.8)	22 (23.9)	2 (25)
ESKD; N (%)	1 (3.7)	15 (16.3)	1 (12.5)
Death; N (%)	3 (11.1)	9 (9.8)	2 (25)
Serious infection; N (%)	4 (14.8)	15 (16.3)	3 (37.5)
Remission, N (%)	20 (71.4)	60 (65.2)	4 (50.0)
**Standard-GC era**	N=10	N=76	N=3
eGFR at biopsy; median (IQR)	38.6 (13.6, 44.0)	19.4 (11.4, 34.5)	8.7 (5.7, 53.9)
ESKD or death; N (%)	2 (20.0)	15 (19.7)	1 (33.3)
ESKD; N (%)	1 (10.0)	10 (13.2)	1 (33.3)
Death; N (%)	1 (10.0)	6 (7.9)	1 (33.3)
Serious infection; N (%)	3 (30.0)	11 (14.5)	2 (66.7)
**Reduced-GC era**	N=17	N=16	N=5
eGFR at biopsy; median (IQR)	15.6 (11.1, 32.1)	19.9 (7.1, 30.9)	10.4 (9.2, 13.3)
ESKD or death; N (%)	2 (11.8)	7 (43.8)	1 (20)
ESKD; N (%)	0 (N/A)	5 (31.3)	0 (N/A)
Death; N (%)	2 (11.8)	3 (18.8)	1 (20)
Serious infection; N (%)	1 (5.9)	4 (25)	1 (20)

Table S2. Outcomes by GC use (in first 12 months), after re-classification of 7 patients

Re-classification	Standard-GC (90)	Reduced-GC (48)	p-value
ESKD or death; N (%)	19 (21.1)	12 (25.0)	0.60
Serious infection; N (%)	17 (18.9)	6 (12.5)	0.33
**Original analysis**	**Standard-GC (97)**	**Reduced-GC (41)**	
ESKD or death; N (%)	21 (21.6)	10 (24.4)	0.72
Serious infection; N (%)	17 (17.5)	6 (14.6)	0.68
